# Organization model, vertical integration, and farmers’ income growth: Empirical evidence from large-scale farmers in Lin’an, China

**DOI:** 10.1371/journal.pone.0252482

**Published:** 2021-06-02

**Authors:** Guiyan Ao, Qiang Liu, Li Qin, Minghao Chen, Shuai Liu, Weiguang Wu

**Affiliations:** 1 School of Economics and Management, Zhejiang A&F University, Hangzhou, Zhejiang, China; 2 School of Public Administration, Guizhou University of Finance and Economics, University Town, Guiyang, Guizhou, China; 3 Research Academy for Rural Revitalization of Zhejiang Province, Zhejiang A&F University, Hangzhou, Zhejiang, China; Institute for Advanced Sustainability Studies, GERMANY

## Abstract

Since China’s reform and opening-up in 1978, the income of rural residents has increased when compared with that of urban residents. However, the income growth rate of farmers is relatively low, and the income gap between urban and rural areas is widening. Using a sample of 1,325 large-scale farming households in Lin’an, this study constructs a theoretical path for how the level of vertical integration and an organization model affect farmers’ income levels and empirically tests the path using a mediation effect analysis model. The results indicate that organization models and vertical integration are important factors that affect farmers’ income levels. The total income and agricultural operation income of farmers who participate in agricultural operation organizations are greater than that of farmers who do not participate in an operation organization. In addition, the total income and agricultural operation income of farmers who produce and process and those who produce, process, and sell are higher than those of farmers who only produce. A farmers’ organization model has both a direct and an indirect positive influence on their income level, with the indirect positive influence coming through the mediating variable of vertical integration. The application of the organizational model can promote the growth of rural households’ total family income and agricultural income by 13.48% and 14.48% respectively, consisting of direct increases of 9.67% and 10.19%, and indirect increases of 3.81% and 4.29% through vertical integration. The results also show that access to credit, agricultural technology training, and the farmer’s education level have significant positive impacts on farming income levels. The findings suggest ways to increase farmers’ income by perfecting agricultural management organization systems, promoting agricultural industrialization, strengthening rural financial support, improving agricultural technical training for farmers, and increasing their level of education.

## 1 Introduction

Improving farmers’ income is the key to solving issues facing agriculture, rural areas, and farmers [1, 2]. China’s reform and opening-up in 1978 has brought great changes to its rural economic development. While the income levels of rural residents, when compared with urban residents, have increased since the reform, the income growth rate of farmers is relatively low, and the income gap between urban and rural areas is widening [3]. Ultimately, increasing farmers’ income is a matter of households’ livelihoods and social stability. Continued steady growth in farmers’ income is crucial to realizing the goal of building a society that is moderately prosperous in all respects. Document No. 1 of the Communist Party of China (CPC) Central Committee in 2018 indicates that safeguarding farmers’ fundamental interests and promoting their common prosperity is the party’s foundation and goal.

Promoting a diversified agricultural organization model and strengthening the vertical integration of agriculture are considered by the government to be important measures to promote the organic integration of small- and large-scale farming and to increase farmers’ income. The vertical integration of agriculture is the coordinated contact method of the successive stages of the production and marketing of agricultural products in the vertical system of the supply chain [4]. Theoretically, increasing the degree of organization of agricultural production and operation, and closely integrating the production, processing, and sales of agricultural products is conducive to achieving vertical integration and thereby improving agricultural production efficiency [5]. Vertical collaboration between farmers and agricultural organizations is conducive to saving transaction costs [6], avoiding market risks [7], maintaining price stability, and ensuring factor support [8, 9], and it has a positive effect on promoting farmers’ income and sustainable agricultural development.

Since 2013, the Chinese government has emphatically promoted the development of new agricultural business entities to increase farmers’ income, mainly through farmer cooperatives, agricultural enterprises, and family farms. They encourage farmers to cooperate with new business entities to increase agricultural income. As of 2018, there were 2.17 million farmer cooperatives legally registered in China, and more than 100 million peasant households had joined the cooperatives, accounting for 49.1% of China’s total peasant households. In 2017, average operating income of each cooperative was about 23.29 million yuan and the distributed profit was nearly 3.38 million yuan [10]. The number of leading agricultural enterprises has climbed to 87,000, and the total income of owner-operated agricultural product processing businesses has exceeded 22 trillion yuan. Farmers increased their total wages and benefits by up to 71.27 billion yuan through cooperative operations with enterprises in 2017 [11]. This indicates that farmers have achieved significant economic benefits through agricultural cooperative operations. In this study, we examine whether we can obtain a similar conclusion by means of a strict econometric model and what the internal impact mechanism of cooperative management on farmers’ income might be. We propose a theoretical basis for scientific decision-making on farmers’ participation in cooperative management and how it can promote the continuous growth of farmers’ income.

Some scholars have studied the impact of cooperative economic organizations on farmers’ incomes. These studies generally affirm that cooperative economic organizations are beneficial to farmers’ income. For example, a study of farming organization models finds that a cooperative economic organization can purchase production resources uniformly and provide standardized production technology to ensure the quantity and quality of agricultural product outputs, increasing farmers’ incomes [12–15]. As an organizational form of scale operations, such organizations can promote specialized agricultural production and, through the principle of reciprocal cooperation, increase peasant household income through capacity building of their members and division of labor within the organization [16, 17]. In addition, these organizations can reduce costs and increase negotiation and bargaining positions, which increase farmers’ income [18].

In terms of industrialization, existing studies show that vertical integration of agriculture can also increase farmers’ income. For example, some scholars examine the action principle between rural industrial structure and farmers’ incomes and quantitatively analyze their degree of correlation. They find that industrialized agricultural operations promote continuous extension of the industrial chain, which promotes employment of the rural surplus labor force, significantly improving peasant household income levels [19, 20]. In contrast, as farmer organizations can help overcome operational risk [21], the lack of these organizations will lessen the ability to overcome natural and market risks, which could result in a decline in farmers’ income levels [22, 23]. Some studies connect small farmers to modern retail outlets through producer organizations, the results showing that vertical and horizontal coordination and value chain management can increase farmers’ income [24–26].

Existing research has laid a strong foundation for this study, but there are also some deficiencies. First, most studies focus on either the organization model or vertical integration in isolation, ignoring the inherent correlation between the two. Second, there are few theoretical analyses of the relevant influence mechanism in existing studies. Moreover, there are few empirical studies based on large samples of farmers, and thus, the reliability of the research conclusions needs to be further verified. To address these gaps, this study examines vertical integration, employs a mediation effect analysis model and empirically tests the influence of vertical integration on organization models and their impact on farmers’ income levels, so as to provide a beneficial reference to help increase farmers’ income.

## 2 Analysis framework

**Fig 1:**
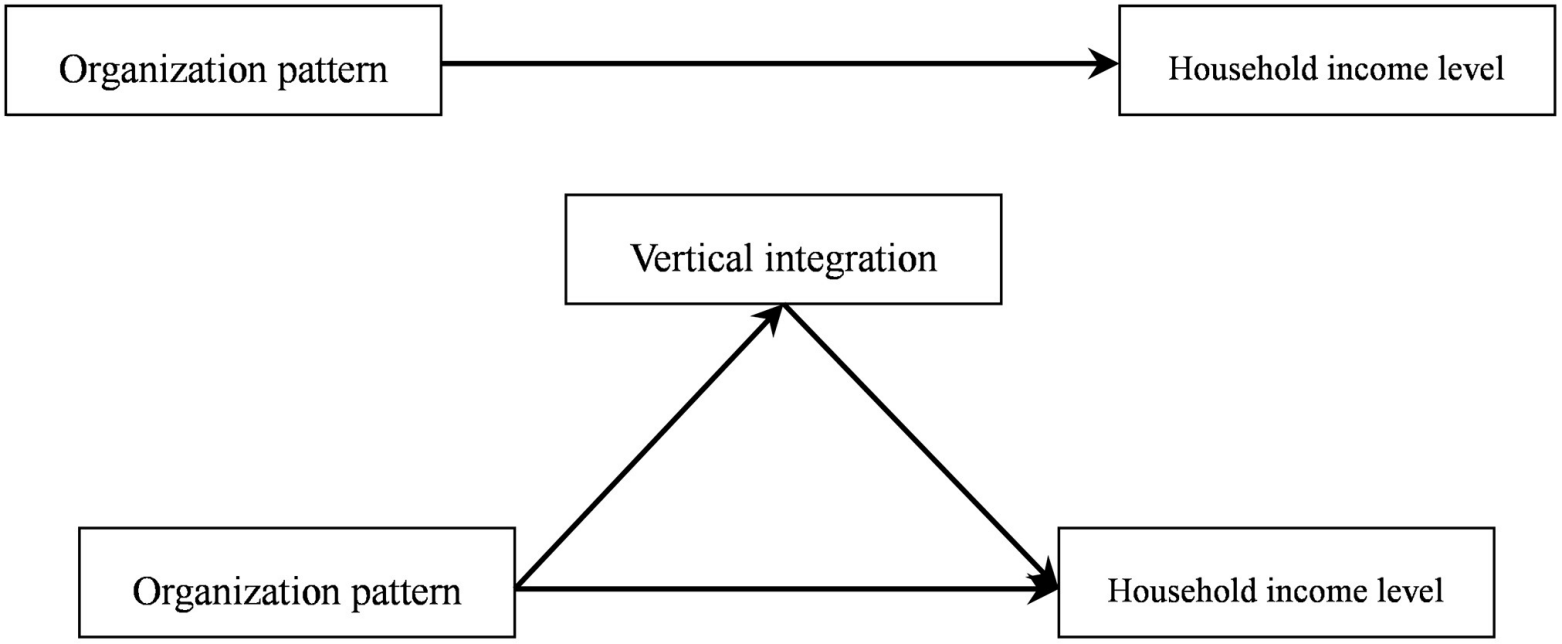
Influence path diagram.

Since the reform and opening-up, China has introduced household contract management in rural areas and a two-tiered management system combining unification. This has, for the most part, liberated productivity in China’s countryside, and farmers have become independent producers and operators. However, the contradiction of small farmers in a large market still exists; most households have no organization and suffer from the disadvantage of a passive position in the market. They not only have to bear the risks of natural disasters and policy, but also face greater market risks. Consequently, since the 1990s, China has begun to encourage new agricultural operations through professional farmers and large family farm cooperatives. Compared with traditional smallholders, the new operators are more organized, vertically integrated, and more closely connected to the market, and more of their total income is derived from agricultural operating income. Based on existing literature [27], this study uses farmers with scale operations to systematically investigate the path through which vertical integration of organization models impact farmers’ income levels (Fig 1) and explores in depth the potential factors that affect farmers’ income levels. Assuming the direct impact of organizational pattern and vertical integration on household income level and the indirect impact of organizational pattern on household income level through vertical integration, the following mediation effect model is constructed:

### 2.1 The influence of organization models on peasant household income levels

Studies have shown that improving the degree of organization among farmers, through, for example, cooperatives, companies, and associations [28, 29], is an important means to increase their income. Companies that work with farmers, peasants’ cooperatives, professional associations, and other new types of agricultural organizations form an external system that incentivizes and constrains the production behavior of peasant households. These organizations establish unified purchases of agricultural products, such as seeds, seedlings, pesticides, and fertilizer for farmers; offer subsidies for the construction of infrastructure; and promote and standardize production technologies; all of which greatly improve farmers’ production environments and their income levels [30]. In addition, organizations can promote division of labor and agricultural specialization in rural areas by developing scale operations, reducing production and transaction costs, and thus, indirectly increasing farmers’ income [31]. Because these various forms of agricultural operation organizations have different institutional arrangements, the hidden incentive and constraint mechanisms differ, which further affects the behavioral efforts of farmers and ultimately leads to differences in their economic performance [32].

### 2.2 The impact of organization models on vertical integration

As cooperatives and companies have a higher degree of industrialization and have more advantages in processing and marketing, when farmers join these organizations, their degree of industrialization will increase correspondingly, and the industrial chain will extend from production to production, processing, and sales [28, 33]. A direct connection between small-scale farm production and big markets has been difficult to draw. Since the mid-1990s, China’s agricultural sector has embarked on a path of industrializing operations, based on the principle of reciprocal cooperation. This approach is operationalized by selecting appropriate agricultural operation organizations, further expanding the market’s scale and division of labor through agricultural specialization and profit-sharing within the organization. However, compared with most developed countries, the level of industrialization of agriculture operations in China is still relatively low. The likely reason is that few farmers in underdeveloped agricultural cooperative economies are organized [34]. Agricultural industrialization management is indispensable for increasing farmers’ organizational development, but it cannot be spontaneously improved. Thus, it is necessary to support and develop various new forms of agricultural operation organizations to increase farmers’ specialization and their degree of organization and thereby to increase the level of agricultural industrialization [35].

### 2.3 The influence of vertical integration on peasant household income levels

Agricultural industrialization, also called “agricultural industry integration,” is described as integrated management of agricultural and industrial production, supply, and marketing, and is the general rule of worldwide agricultural development [36]. Increase in the degree of vertical integration can improve production efficiency, disperse agricultural production risks, and improve the income distribution of farmers in different production processes, thereby increasing their agricultural operation income and total family income [33, 37–39]. Advancing agricultural industrialization is conducive to extending the processing industry chain for agricultural products, and increasing the stability of agricultural product sales channels greatly improves the level of agricultural product market integration, further boosting farmers’ income levels [40]. With the continuing increase in demand for agricultural products and the extension of the industrial chain, agricultural industrialization has gradually become an effective way to solve the problem of rural surplus labor employment and increase farmers’ income.

To summarize, different agricultural organization models have dissimilar effects on farmers’ behavioral efforts, which can directly impact their income levels. In addition, agricultural organizations may indirectly affect farmers’ income levels by promoting agricultural industrialization. This paper proposes the following theoretical hypotheses:

**H1**: The organizational model has a positive effect on increasing farmers’ income.
**H2**: The organizational model promotes the vertical integration of farmers, thereby indirectly increasing farmers’ income.

## 3 Data sources and model settings

### 3.1 Sample profile and data sources

Lin’an, a typical agricultural county in China, is located northwest of the economically developed Zhejiang Province. In 2018, 61% of Lin’an’s population was agricultural and the per capita disposable income of rural residents was 25,598 yuan. The province has nine distinctive and profitable agricultural industries, including bamboo shoots, hickory nuts, and others; the value of the annual production of bamboo shoots and hickory nuts alone is 4.8 billion yuan. The vertical integration of Lin’an’s agricultural and farmers’ organizations is high, and agricultural cooperation is well organized, with integrated production, supply, and marketing. Organizational models such as “agricultural company + farmers,” “farmer cooperative + farmers,” and “agricultural company + farmer cooperative + farmers” play an important role in improving farmers’ incomes. Using farmers in Lin’an to study the impact of vertical integration and organization models on peasant household income can provide significant insights to aid in China’s rural revitalization and industrial poverty alleviation.

The study uses the third agricultural census data from Lin’an’s agricultural scale enterprises and includes households whose annual income is mainly from agricultural production and operations and is 100,000 yuan or more. The data are cross-sectional data accurate as of December 31, 2016 and cover Lin’an’s 13 townships, comprising 1,325 farmers. The sample’s main statistical indicators include individual characteristics (gender, age, and education of household head), household characteristics (size, average age, average education of the household’s labor force, and household income level), and management features (participation in agricultural operation organizations, production and business operation modes, credit acquisition, land scale, and planting of cash crops).

The characteristics of how farmers are organized and their level of vertical integration can be measured by the agricultural operation organizations in which they participate and the production and operation modes they adopt. Based on the situation in Lin’an, a comparative analysis is performed of two forms of agricultural operation organizations, “professional association + farmers” and “farmer cooperative + farmers”; as well as two production and operation models, “production and processing” and “production, processing, and sales.”

“Professional association + farmers” agricultural management organizations in Lin’an are mainly involved in bamboo, hickory, and other industries of professional economic associations. All professional associations are non-profit organizations whose members are mainly agricultural operating units and farmers engaged in work related to production and operations related to agricultural products. The income of the associations can only be used to develop the association, and cannot be distributed among members. Their primary function is to provide farmers with unified technical guidance and training, before, during, and after production, and with unified sales of agricultural products to improve farmers’ self-management levels. “Farmer cooperative + farmers” organizations in Lin’an are mainly professional cooperatives for industries such as bamboo shoots, hickory, and others. All cooperatives are non-profit organizations, with farmers comprising over 80% of the membership. In addition to providing technical training for members and a marketing channel for agricultural products, these cooperatives seek to obtain economic benefits by developing agricultural production and members, in accordance with the amount and distribution of surplus, which is more beneficial for primary producers.

Lin’an mainly processes bamboo shoots, hickory nuts, and other agricultural products, with the economy in the process of gradually transforming from primary processing to refined processing. “Production and processing” refer to the production and operations by which farmers sell their agricultural products to distributors after primary processing; distributors then process these products and sell them to end consumers. “The biggest food bamboo garden in South China” is a motto that articulates Lin’an’s advantages in its bamboo industry development. However, some farmers fail to master the fine processing technology, and continue to treat their own bamboo shoots as primary agricultural products without processing or with simple processing, and sell them directly. Therefore, under the “production and processing” operating mode, farmers’ industrial chains end after the initially processed agricultural products are sold to distributors. The “production, processing, and sales” mode of production and operation refers to farmers who, on the premise of mastering certain processing technologies, carry out the fine processing of the agricultural products produced or create their own brand of agricultural products and directly sell them to end consumers. For example, as the largest hickory processing and sales center in China, Lin’an has distributed its hickory sales outlets to over 20 provinces (cities) by developing regional brands. Local farmers can process their own hickory and sell it directly to end consumers through these outlets. Therefore, the operation mode of “production, processing, and sales” extends farmers’ industrial chains from the primary producer to the end consumer.

Table 1 describes the basic characteristics of the sample farmers and the differences in farmers’ income levels based on household characteristics (vertical integration and organization model).

**Table 1:** Differences in sample farmers’ incomes by household characteristics.

Characteristics of peasant household	Options	Number	Percentage (%)	Household income (ten thousand yuan)	Agricultural operation income (ten thousand yuan)
Categories of participation in agricultural operation organizations	No participation	1143	86.26	26.16	22.65
	Professional association + farmers	46	3.47	51.61	46.71
	Farmer cooperative + farmers	100	7.55	44.15	40.96
	Agricultural company + cooperative + farmers	36	2.72	57.64	53.90
Types of production and operation methods adopted	Production	1082	81.66	25.69	22.08
	Production and processing	131	9.89	43.54	40.85
	Production, processing, and sales	112	8.45	46.97	43.21
Gender of household head	Woman	100	7.55	27.68	23.62
	Man	1225	92.45	29.38	25.89
Age of household head	Under 40 years old	74	5.58	28.56	25.82
	40–60 years old	977	73.74	23.53	22.97
	> 60	274	20.68	29.28	25.71
Years of schooling of household head	0–6 years	384	28.98	24.62	21.44
	7–9 years	703	53.06	27.51	24.10
	10–12 years	210	15.85	41.89	37.52
	13–16 years	28	2.11	41.87	36.46
Labor force number	1–2	347	26.19	25.31	22.98
	3–4	928	70.04	30.45	26.36
	>5	50	3.77	34.56	32.84
Average age of the workforce	16–39 years old	437	32.98	31.66	27.91
	40–60 years old	831	62.72	28.67	25.13
	61–65 years old	57	4.30	19.39	18.06
Average years of education of the labor force	<6 years	84	6.34	18.16	16.93
	7–9 years	477	36.00	25.69	22.61
	10–12 years	642	48.45	31.15	27.29
	>13 years	122	9.21	40.90	35.65
Population burden coefficient	<0.3	852	64.30	29.14	25.57
	0.3–0.6	311	23.47	29.37	25.86
	>0.6	162	12.23	29.63	26.20
Agricultural technology training	<0.3	1085	81.89	26.22	22.74
	0.3–0.6	161	12.15	41.39	37.30
	>0.6	79	5.96	46.12	42.93
Percentage of non-farm employment	<30	253	19.09	30.21	29.95
	30–60	487	36.75	26.54	23.01
	>60	585	44.15	31.10	26.14
Whether there are civil servants	No	1271	95.92	29.00	25.57
	Yes	54	4.08	35.17	29.17
Main types of agricultural occupations	Planting industry	887	66.94	24.11	20.56
	Forestry	171	12.91	26.10	22.25
	Animal husbandry	256	19.32	47.99	44.71
	Fishery	11	0.83	56.67	53.03
Access to credit	No	1108	83.62	26.67	23.14
	Yes	217	16.38	42.44	38.90
Operating land area	Under 5 Mu	1129	85.21	27.93	24.21
	5–10 Mu	69	5.21	31.76	29.60
	Above 10 Mu	127	9.58	39.69	37.02
Transferred land area	<5 Mu	1166	88.00	27.42	23.79
	5–10 Mu	79	5.96	33.09	30.34
	>10 Mu	80	6.04	52.12	49.16
Cash crops are planted	No	943	71.17	27.12	23.40
	Yes	382	28.83	34.52	31.45

Note: Statistics based on survey data.

Table 1 reports the characteristics of both organization models and vertical integration. The willingness of peasant households to participate in agricultural operation organizations is low (i.e., less than 15% of peasant households). The proportions of farmers in “professional association + farmers,” “farmer cooperative + farmers,” and “agricultural company + farmer cooperative + farmers” who participate range from 2.72% to 7.55%. Compared to farmers who do not participate in any agricultural operation organization, farmers who participate in the other categories have relatively higher incomes; the total income and agricultural income of these farmers are higher than that of other farmers by at least 68.77% and 80.84%, respectively. Vertical integration of peasant households is generally low, as production is the primary mode of production and operation, accounting for more than 80% of the sample. When compared to farmers with less vertical integration (production farmers), farmers with more vertical integration (production and processing farmers) have higher income; their total income and agricultural income increased by at least 69.48% and 85.01%, respectively.

Table 1 also describes the farmers’ individual characteristics. More than 90% of household heads in the sample are males, and they tend to be middle-aged or elderly. Their education level is generally low, concentrated at the junior high school level and below; less than 20% of the farmers attended high school and received 12 years of education. In addition, the data show that as the household head’s educational level increases, the household income level also increases.

Table 1 also presents some characteristics of farmers’ households, such as the labor force number per household; over 70% of households have three or four laborers. The most common (over 60%) average age of the household labor force is 40 to 60 years old. The average education level of more than 90% of household labor forces is high school, with no more than 13 years of education. The results also show that the greater the size of the household’s labor force and the higher the number of years of education, the higher the household’s income level. Moreover, the higher the labor force’s average age, the lower the household’s income level. In addition, the population burden coefficient, that is, the ratio of non-working population to working population, is less than 0.3 (which represents over 60% of households); its correlation with farmers’ income is not apparent. There is a clear positive correlation between participation in agricultural technology training and income level (in as many as 80.21% of households); the average number of times participating in labor training is less than 0.3 times per household. A higher rate of non-farm employment increases the total family income and accounts for more than 30% of the overall increase. There is a clear position between whether there are civil servants in the household and the farmer’s income; however, only 4.08% of households have civil servants in the household.

The characteristics of peasant household operations reflect that different types of agricultural occupations affect income levels differently. The planting industry has the least impact on income, and fishery has the greatest impact. It can be seen from the table that the agricultural and total incomes of households with credit are higher than those of households without credit, while rural financial support is relatively weak, as the proportion of farmers who can obtain credit is only 16.38%. The larger the scale of land operation, the higher the level of household agricultural and total incomes. However, the operating scale of peasant households is generally small, with less than 10% of the actual farmland area being more than 10 mu; there are similar characteristics for transferred land. In addition, the agricultural and total incomes of households with cash crops are higher than those for households without cash crops; about 30% of peasant households plant cash crops.

The analysis shows that there is a certain correlation between farmers’ income levels and factors such as organization model and vertical integration. However, determining whether there is a causal relationship requires econometric analysis. Therefore, this study builds an econometric analysis model to empirically analyze how factors such as vertical integration and organization model impact farmers’ income levels.

### 3.2 Empirical model construction and variable selection

In this study, total household income and agricultural operating income are selected to measure farmers’ income levels. First, based on the function that determines farmers’ incomes [41], an econometric equation is established and expanded to empirically analyze how different forms of organization and different levels of industrialization affect farmers’ income levels. Then, a theoretical path for the influences among organization form, vertical integration, and farmers’ income levels is constructed and a mediating effect analysis model is used to empirically verify the path constructed. The specific model is as follows:

$${{{lny=\beta }_{0}+\gamma }_{1}Organize+\gamma }_{2}industry+\sum_{i}\beta_{i}x_{i}+\varepsilon$$ (1)

Eq (1) is a farmers’ income determination equation model. In the equation, *y* is the farmer’s income level (total household income or agricultural business income); *organize* represents the different forms of organization models, and *industry* represents different levels of vertical industrialization. *x*_*i*_ represents the control variables, which include individual characteristics (household head gender, age, years of education, etc.), household characteristics (labor force number, average age, average years of education, burden coefficient of the household labor force, etc.), operation characteristics (main types of agricultural occupations, access to credit, land management scale, whether the household plants cash crops, etc.), and regional dummy variables. In addition, *γ* and *β* are the parameters to be estimated, and ε is a random error term.

$${{{lny=\alpha }_{0}+\alpha }_{1}Organize}_{}+\sum_{i}\alpha_{i}x_{i}+\varepsilon$$ (2)

$${{{industry=\beta }_{0}+\beta }_{1}Organize}_{}+\sum_{i}\beta_{i}x_{i}+\mu$$ (3)

$${{{lny=\gamma }_{0}+\gamma }_{1}Organize+\gamma }_{2}industry+\sum_{i}\gamma_{i}x_{i}+\varphi$$ (4)

Eqs (2)–(4) are mediating effect analysis models. In Eq (2), *α*_1_ represents the total effect of the organization model on farmers’ income levels; *β*_1_ indicates the effect of the *organization model on vertical integration; γ*_1_ indicates the direct effect of the organization model on farmers’ income levels after controlling for *the influence of vertical integration; γ*_2_ indicates the effect of vertical integration on farmers’ income levels after controlling for the influence of the organization model. The coefficient product *β*_1_*γ*_2_ represents the effect of the organization model on farmers’ income levels through vertical integration, that is, the indirect effect. The following relationship exists among the three effects: *α*_1_ = *γ*_1_+*β*_1_*γ*_2_. That is, the total effect is equal to the sum of the direct and indirect effects. In addition, *x*_*i*_ represents other control variables that affect the degree of organization or farmers’ income levels; and *ε*, *μ*, and *φ* are random error terms. The specific variable names and descriptions are shown in Table 2.

**Table 2:** Variable names and descriptions.

	Variable name	Variable description	Unit / evaluation	Mean value	Standard deviation
Dependent variable	y_1_	Household income	Ten thousand yuan	29.26	22.86
	y_2_	Agricultural operation income	Ten thousand yuan	25.72	22.28
Organization model	organize	Categories of participation in agricultural operation organizations	1 = no participation	1.27	0.71
			2 = professional association + farmers		
			3 = farmer cooperative + farmers		
			4 = agricultural company + farmer cooperative + farmers		
Vertical integration	industry	Types of production and operation methods adopted	1 = production	1.27	0.60
			2 = production and processing		
			3 = production, processing, and sales		
Household head characteristics	x_1_	Gender of household head	0 = woman; 1 = man	0.92	0.26
	x_2_	Age of household head	years	53.49	8.67
	x_3_	Years of schooling of household head	years	8.70	2.38
Household characteristics	x_4_	Labor force number	person	3.03	0.95
	x_5_	Average age of labor force	years	43.57	7.61
	x_6_	Average years of education of labor force	years	9.94	2.01
	x_7_	Population burden coefficient	Number of non-laborers/ number of laborers	0.25	0.36
	x_8_	Agricultural technology training	Proportion of labor force trained in agricultural technology	0.11	0.24
	x_9_	Percentage of non-farm employment	Non-agricultural labor force number/ agricultural labor force number	0.55	0.35
	x_10_	Whether there are civil servants	0 = no; 1 = yes	0.04	0.20
Operation characteristics	x_11_	Main types of agricultural occupations	1 = planting industry	1.54	0.84
			2 = forestry; 3 = animal husbandry; 4 = fishery		
	x_12_	Access to credit	0 = lack of credit; 1 = Getting credit	0.16	0.37
	x_13_	Operating land area	Mu	7.28	29.79
	x_14_	Transferred land area	Mu	0.08	0.40
	x_15_	Cash crops are planted	0 = no; 1 = yes	0.29	0.45

#### 3.2.1 Household income

In the analysis, total household income and agricultural operation income are used to represent the income variables. For the large-scale farmers, the total household income and agricultural operation income are 292,600 yuan and 257,200 yuan respectively. Agricultural operating income thus accounts for 87.90% of the total.

#### 3.2.2 Organization model

The categories of participation in agricultural operation organizations are used to represent the organizational model variable. “No participation” is set as the control group and assigned a value of 1; the “farm + association” model is assigned a value of 2. In general, the degree of organization of farmers is not high, with an average of 1.27, and most farmers are only in the relatively low-level organizational model of “professional association + farmers.”

#### 3.2.3 Vertical integration

The types of production and operation methods are adopted to represent the vertical integration variable. “Production” is set as the control group and assigned a value of 1. “Production and processing” is then assigned a value of 2, and “production, processing, and sales” is assigned a value of 3. In general, the degree of vertical integration of farmers is not high, with an average of 1.27, and there are few production, processing, and sales farmers.

Following previous studies [42–44], the following control variables are also selected in the empirical model.

#### 3.2.4 Household head characteristics

The household head’s gender, age, and years of schooling are selected as household head characteristics. The household head’s gender may affect household decision-making and thus household income. In addition, the older farmers are, the more difficult it is for them to adapt to the new era of development needs, which might lead to making certain household decisions that fail to increase household income. However, the longer farmers attend school, the more human capital they accumulate, which can lead to an increase in household income level.

#### 3.2.5 Household characteristics

Households with a large labor force have more people engaged in agricultural production and management or non-agricultural work, promoting growth in the households’ income. By contrast, a labor force of higher average age is less physically fit, reducing the likelihood and amount that they will engage in production and business activities, in turn resulting in lower household income. If the labor force’s average years of education is higher, it may lead to a higher level of agricultural or off-farm work, resulting in higher household income levels. However, the population burden coefficient may hinder this growth. Agricultural technology training helps break the bottlenecks of capital and labor in agricultural production and operation, thus raising farmers’ production and incomes. Non-agricultural labor income is generally higher than that of agriculture; the higher the rate of non-farm employment, the more beneficial it is for family income. Social capital accumulation, such as family members working as civil servants, leads to higher peasant household income, because capital is an important factor for promoting production and operation.

#### 3.2.6 Operation characteristics

The main agricultural occupations that farmers engage in will also affect their income. If loans can be obtained through private lending or formal bank credit, higher investment returns are more likely, thus improving household income. Land is one of the key factors in agricultural production and management: the larger the land area owned by peasant households, the more likely they are to carry out scale operations and thus receive a reward of corresponding scale. The transfer of land in or out will affect the scale of land operation and thus also affect income. Compared with food crops, the economic value of cash crops is relatively higher, so it is very likely that farmers who plant cash crops will have higher incomes than farmers who do not.

## 4 Empirical results and analysis

### 4.1 Influence of organization model and vertical integration on farmers’ income levels

Based on the equation that determines farmers’ income, this study empirically analyzes the impact of different organization model forms and different degrees of industrialization on farmers’ income levels. The results of the model estimation are shown in Table 3. The F values for both models are significant at the 1% level, indicating that the models have good fit and can be used to interpret and analyze the data. Moreover, the mean VIF is 1.46, showing that the problem of multicollinearity is not serious. The estimation results can then be used for subsequent analysis.

**Table 3:** Influence of organization model and vertical integration on farmers’ income levels.

Explanatory variable		Model 1 y = ln(Household income)	Model 2 y = ln(Agricultural operation income)
Organization model	Professional association+ farmers	0.1322*	0.1462*
	Farmer cooperative+ farmers	0.1606***	0.1735***
	Agricultural company + farmer cooperative + farmers	0.2770***	0.2855***
Vertical integration	Integration of production and processing	0.2991***	0.3252***
	Integration of production, processing and sales	0.3421***	0.3906***
Household head characteristics	Gender of household head	-0.0830	-0.0652
	Age of household head	-0.0009	0.0002
	Years of schooling of household head	0.0383***	0.0492***
Household characteristics	Labor force number	0.0552***	0.0465***
	Average age of labor force	-0.0046	-0.0053
	Average years of education of labor force	0.0307***	0.0173
	Population burden coefficient	0.0015	0.0412
	Agricultural technology training	0.1726***	0.1471**
	Percentage of non-farm employment	0.1429***	-0.0424
	Whether there are civil servants	0.1506**	0.1460**
Operation characteristics	Main types of agricultural occupations	0.1531***	0.1734***
	Access to credit	0.1381***	0.1575***
	Operating land area	0.0032***	0.0039***
	Transferred land area	0.0299	0.0194
	Cash crops are planted	0.0935***	0.0949**
Constant term		2.3635***	2.3407***
Dummy variables of villages and towns		Control	Control
Adjusted R^2^		0.4515	0.4659
F value		29.68***	31.37***

Note:

***, ** and * represent significance at the 1%, 5%, and 10% level, respectively.

Model 1 examines how factors such as vertical integration and organization model impact household income, while model 2 examines the impact of these factors on agricultural operations income. The estimation results show that there are no observable differences between the results of model 1 and model 2 in the significance level or sign of the coefficients, indicating that the estimation results are robust.

#### 4.1.1 The influence of organization model on farmers’ household income levels

According to the model estimation results, the influence of all three organizational models, including “professional association,” “farmer cooperative,” and “company + farmer cooperative + farmers,” have a significant positive impact on farmers’ agricultural income and total family income, confirming hypothesis H1. The estimated coefficient shows that, when compared with farmers who did not participate in an operation organization, the total household income of farmers who participated in “professional association,” “farmer cooperative,” and “company + farmer cooperative + farmers” increased from 13.22% to 27.70%, while the increase in agricultural operation income ranged from 14.62% to 28.55%. The increase in farmers’ income levels differ depending on the organization model. “Company + farmer cooperative + farmers” is the highest, followed by “farmer cooperative,” and finally “professional association.” Moreover, the same organization model has different effects on different types of farming income, and each organization model has a larger improvement effect on farmers’ agricultural operation income than on total income.

#### 4.1.2 The influence of vertical integration on farmers’ household income levels

The model estimation results also show that the effects of the two production and operation modes on total household income are significantly positive. Thus, compared to production-oriented farmers, both total household income and agricultural operation income are higher for farmers whose production and operations are integrated (“production and processing” and “production, processing, and sales”). Similarly, there are differences in how the level of industrialization affects farmers’ incomes. The effect of production, processing, and marketing integration is greater than that of production and marketing integration. Further, there are differences in the effects of the same mode of production and operation on different income types. Each mode of production and operation has a larger role in increasing farmers’ agricultural income compared to total income.

#### 4.1.3 The influence of other factors on farmers’ income levels

The estimated results show that the household head’s years of schooling, the number of household members in the labor force, their average years of education, their amount of agricultural technology training, whether they include civil servants, their main types of agricultural occupations, access to credit, operating land area, and whether cash crops are planted can all significantly improve their total household income and agricultural income, while the household head’s gender and age, the average age of the labor force, the population burden coefficient, and transferred land area do not significantly affect farmers’ income levels.

### 4.2 The mediation effect test

This study constructs an influence path among vertical integration, organization model, and farmers’ income level. A mediating effect analysis model is used to empirically test the path constructed to determine whether longitudinal integration has a mediation effect; in other words, it is necessary to test whether the path of the theoretical model of the mediation effect is reasonable [45]. Therefore, a new mediation effect test method is adopted in this study, examining the mediation effect of vertical integration. The specific process is as follows.

The first step is to construct a model in which the household’s total income is the explained variable and the organization model and control variables are included as explanatory variables (model 3). If the coefficient of the organization model is significant, it will be argued that the organization model has a mediating effect; otherwise, it will be argued that there is a masking effect. The second step is to construct a model in which vertical integration is the explained variable and the organization model and control variables are included as explanatory variables (model 4). Next, in model 5, the household’s total income is the explained variable, and the organization model, intermediating variables, vertical integration, and control variables are included as explanatory variables. If the coefficient of the organization model in model 4 and the coefficient of longitudinal integration in model 5 are both significant, then the indirect effect is also significant. If at least one is not significant, proceed to step 3; otherwise, skip step 3 and go to step 4. In step 3, a bootstrap method is used to test the null hypothesis that the product of the coefficient of the organization model in model 4 and the coefficient of the longitudinal integration in model 5 = 0. If significant, then the indirect effect is significant—continue to the fourth step; otherwise, stop testing. The fourth step tests whether the coefficient of the organization model in model 5 is significant. If so, the direct effect is significant—continue to the fifth step; otherwise, stop testing, and conclude that the longitudinal integration has a complete mediating effect. The fifth step compares the sign of the coefficient of the organization model in model 5 to the sign of the product of the coefficient of the organization model in model 4 and the coefficient of the longitudinal integration in model 5. If the signs are the same, the vertical integration has a partial mediating effect, and if different, a masking effect.

The main purpose of this analysis process is to examine whether vertical integration plays a mediating role in the organization model’s influence on household income. Similarly, using the same explanatory and control variables, household income as the explained variable is replaced by agricultural operation income in models 6 and 7. The same testing procedure is used to test whether vertical integration plays a mediating role in the organization model’s influence on farmers’ agricultural operation income.

The test results are shown in Table 4. The F values of models 3, 4, 5, 6 and 7 are significant at the 1% level, indicating that the models’ overall ability to interpret the estimated results is strong and they can be used to interpret and analyze the data.

**Table 4:** Mediation effect test between vertical integration and organizational model and total household income and agricultural income.

Explanatory variable	Model 3 y = ln (Household income)	Model 4 y = Vertical integration	Model 5 y = ln (Household income)	Model 6 y = ln (Agricultural operation income)	Model 7 y = ln (Agricultural operation income)
Organization model	0.1348***	0.1974***	0.0967***	0.1448***	0.1019***
Vertical integration			0.1930***		0.2175***
Miscellaneous variables	Control	Control	Control	Control	Control
Constant term	2.3348***	1.4146***	2.0618***	2.3191***	2.0098***
Adjusted R^2^	0.4277	0.3116	0.4499	0.4386	0.4644
F value	30.10***	18.63***	31.93***	31.40***	33.78***

Note:

***, **and * represent significance at the 1%, 5%, and 10% levels, respectively; the “miscellaneous variables” same as model 1.

In the tests of the mediating effect, first, the organization model is tested to see whether it has a significant influence on total household income and agricultural operation income as the explained variables. In both models 3 and 6, the coefficients of the organization model are significant at the 1% level, indicating that the organization model has a positive impact on farmers’ income levels. Second, the organization model is tested to see if it has a significant influence on longitudinal integration. According to model 4’s estimation results, the coefficient of the organization model where longitudinal integration is the dependent variable is significant at the 1% level, indicating that the organization model promotes improvement in farmers’ vertical integration. Finally, a mediation effect test is conducted based on the estimates in models 5 and 7. After vertical integration, the mediating variables of farm household income and agricultural management organization model are introduced and are significant at the 1% level; moreover, the vertical integration of farmers’ household income and the impact of agricultural management income are also positive and significant at the 1% level. Combined with the above model estimation results, this indicates that vertical integration plays a partial mediating role in the influence of the organization model on total household income and agricultural operation income, verifying hypothesis H2.

Having verified the rationale of the path established based on the mediation effect theory, we now calculate values of the effects of the organization model on total household income and agricultural operation income. According to the results, the organizational model can promote the growth of rural households’ total family income and agricultural income by 13.48% and 14.48%, respectively, consisting of a direct increase to income of 9.67% and 10.19% and an indirect increase of 3.81% and 4.29% through vertical integration. The indirect effects account for 28.26% and 29.63% of the total effects. This shows that the mediating transmission mechanism of vertical integration is highly significant to explain the organization model’s impact on farmers’ income levels.

## 5 Conclusion and discussion

### 5.1 Conclusion and implications

Using 1,325 peasant households in Lin’an as its sample, this study applies an econometric model to empirically analyze the impact of organization model, vertical integration, and other factors on farmers’ household income levels. By establishing an organization model incorporating vertical integration and farmers’ income levels, among the three influence paths, we construct a path for empirical testing using a mediation effect analysis model. On this basis, we offer the following conclusions and suggestions.

First, the organization model is an important factor that affects farmers’ income levels. Compared to farmers who did not participate in an organization, those who did had better coordination. The incentives and restrictions of the organization system can improve the degree of behavioral effort, which in turn increases farmers’ income levels. In addition, the degree of institutional incentives and constraints in organization models differs, and they have different effects on promoting growth in farmers’ incomes. The government should actively create conditions to encourage and guide farmers to participate in the various types of agricultural management organizations, by establishing an organization to promote its members’ interests, as a linking mechanism to promote increases in farmers’ income.

Second, vertical integration is another key factor that affects farmers’ income levels. Compared with production-oriented farmers, “production and processing-oriented” and “production, processing, and sales-oriented” farmers have improved vertical integration, their industrial chain has been extended, there is more in-depth processing of agricultural products, and the distribution channels for agricultural products are more stable, thus increasing farmers’ incomes. In addition, the organization model affects farmers’ income levels not only directly but also indirectly, their income through the mediating variable of vertical integration. Therefore, the government should give full consideration to the role of agricultural operation organizations in promoting agricultural industrialization, supporting and developing various forms of agricultural operation organizations, and promoting the level of agricultural industrialization operations through organizations to improve farmers’ income levels.

Third, as a means of rural financing to support agricultural development, agricultural loans play an important role in increasing agricultural production intensity and farmers’ incomes. Agricultural funds are essential for agricultural production and operations. However, the direct financing system for agricultural production in China needs improvement. Moreover, agricultural producers’ capacity to accumulate funds is quite limited, which makes the availability of the funds needed for agricultural production largely dependent on financial institutions. To bolster financial support for the development of modern agriculture, the government can formulate corresponding preferential tax policies for agriculture-related loans to stimulate financial institutions to return to the rural financial markets.

Fourth, education level and agricultural technology training have significantly positive impacts on farmers’ income levels. Therefore, improving farmers’ education levels and strengthening training in agricultural technology will help increase their income. The quality of human capital largely determines farmers’ household income. The educational level of those currently involved in production and operations is generally not high, and their enthusiasm for participating in agricultural technical training is generally low; these characteristics seriously hinder the development of modern agriculture and greatly constrain improvement in farmers’ household incomes. The government should therefore strengthen agricultural technical training and education for farmers, to promote the transformation of traditional farmers into new, professional farmers.

### 5.2 Limitations and future research suggestions

It should be noted that the data used in this research are cross-sectional, and the level of organization and vertical integration in farmers’ households is a dynamic, changing process. The influence of organization models and vertical integration on farmers’ household incomes also undergoes dynamic adjustment, and may vary from period to period. However, according to the theoretical analysis provided and empirical tests referred to in this study, the significant influence mechanism identified here should exist; more accurate mediation effects can be obtained if the data allow.

In addition, this study only considered Lin’an, a typical area in East China, as a case for study, and therefore the generalizability of its conclusions is limited. Lin’an used to be a typical poor mountainous county. However, after China’s reform and opening-up, Lin’an took the lead in developing agricultural zoning and was set on the agricultural development road characterized by agricultural industrialization and management cooperation. Indeed, it has developed from a poor mountainous county into one of the top 100 economically and socially developed counties in China. The per capita net income of farmers increased dramatically from 176 yuan in 1978 to 25,598 yuan in 2018. It was selected as one of the “Top 100 National Comprehensive Strength Counties,” which represent the epitome of China’s rapid agricultural economic development relying on cooperative operations. Nevertheless, its representativeness is still insufficient; in the future, the samples can be expanded to more regions of China, and international comparative studies can also be conducted to comprehensively verify the research conclusions, making them more widely applicable and valuable.

## Supporting information

S1 Data (XLSX)
